# Changes and Inequities in Adult Mental Health–Related Emergency Department Visits During the COVID-19 Pandemic in the US

**DOI:** 10.1001/jamapsychiatry.2022.0164

**Published:** 2022-03-16

**Authors:** Kayla N. Anderson, Lakshmi Radhakrishnan, Rashon I. Lane, Michael Sheppard, Jourdan DeVies, Roseric Azondekon, Amanda R. Smith, Rebecca H. Bitsko, Kathleen P. Hartnett, Barbara Lopes-Cardozo, Rebecca T. Leeb, Katharina L. van Santen, Kelly Carey, Sophia Crossen, Taylor P. Dias, Sam Wotiz, Jennifer Adjemian, Loren Rodgers, Rashid Njai, Craig Thomas

**Affiliations:** 1 National Center for Injury Prevention and Control, US Centers for Disease Control and Prevention, Atlanta, Georgia; 2 Center for Surveillance, Epidemiology, and Laboratory Services, US Centers for Disease Control and Prevention, Atlanta, Georgia; 3 National Center for Chronic Disease Prevention and Health Promotion, US Centers for Disease Control and Prevention, Atlanta, Georgia; 4 Oak Ridge Institute for Science and Education, Oak Ridge, Tennessee; 5 Epidemic Intelligence Service, US Centers for Disease Control and Prevention, Atlanta, Georgia; 6 National Center on Birth Defects and Developmental Disabilities, US Centers for Disease Control and Prevention, Atlanta, Georgia; 7 Center for Global Health, US Centers for Disease Control and Prevention, Atlanta, Georgia; 8 ICF International, Atlanta, Georgia; 9 InductiveHealth Informatics, Ottawa, Kansas; 10 Deloitte, Atlanta, Georgia; 11 Office of Minority Health and Health Equity, US Centers for Disease Control and Prevention, Atlanta, Georgia

## Abstract

**Importance:**

The COVID-19 pandemic has negatively affected adult mental health (MH), with racial and ethnic minoritized groups disproportionately affected.

**Objective:**

To examine changes in adult MH-related emergency department (ED) visits into the Delta variant pandemic period and identify changes and inequities in these visits before and during COVID-19 case surges.

**Design, Setting, and Participants:**

This epidemiologic cross-sectional study used National Syndromic Surveillance Program data from US adults aged 18 to 64 years from 1970 to 2352 ED facilities from January 1, 2019, to August 14, 2021. All MH-related ED visits and visits related to 10 disorders (ie, anxiety, depressive, bipolar, schizophrenia spectrum, trauma- and stressor-related, attention-deficit/hyperactivity, disruptive behavioral and impulse, obsessive-compulsive, eating, and tic disorders) were identified.

**Exposures:**

The following periods of MH-related ED visits were compared: (1) high Delta variant circulation (July 18-August 14, 2021) with a pre-Delta period (April 18-May 15, 2021), (2) after a COVID-19 case peak (February 14-March 13, 2021) with during a peak (December 27, 2020-January 23, 2021), and (3) the Delta period and the period after a COVID-19 case peak with the respective corresponding weeks during the prepandemic period.

**Main Outcomes and Measures:**

ED visits for 10 mental disorders and all MH-related visits.

**Results:**

This cross-sectional study included 107 761 319 ED visits among adults aged 18 to 64 years (59 870 475 [56%] women) from January 1, 2019, to August 14, 2021. There was stability in most MH-related ED visit counts between the Delta and pre-Delta periods (percentage change, −1.4% to −7.5%), except for eating disorders (−11.9%) and tic disorders (−19.8%) and after a COVID-19 case peak compared with during a peak (0.6%-7.4%). Most MH-related ED visit counts declined in the Delta period relative to the prepandemic period (−6.4% to −30.7%); there were fluctuations by disorder when comparing after a COVID-19 case peak with the corresponding prepandemic period (−15.4% to 11.3%). Accounting for ED visit volume, MH-related ED visits were a smaller proportion of visits in the Delta period compared with the pre-Delta period (visit ratio, 0.86; 95% CI, 0.85-0.86) and prepandemic period (visit ratio, 0.80; 95% CI, 0.79-0.80). After a COVID-19 case peak, MH-related ED visits were a larger proportion of ED visits compared with during a peak (visit ratio, 1.04; 95% CI, 1.03-1.04) and the corresponding prepandemic period (visit ratio, 1.11; 95% CI, 1.11-1.12). Of the 2 510 744 ED visits included in the race and ethnicity analysis, 24 592 (1%) were American Indian or Alaska Native persons, 33 697 (1%) were Asian persons, 494 198 (20%) were Black persons, 389 740 (16%) were Hispanic persons, 5000 (0.2%) were Native Hawaiian or Other Pacific Islander persons, and 1 172 683 (47%) were White persons. There was between- and within-group variation in ED visits by race and ethnicity and increases in selected disorders after COVID-19 peaks for adults aged 18 to 24 years.

**Conclusions and Relevance:**

Results of this cross-sectional study suggest that EDs may have increases in MH-related visits after COVID-19 surges, specifically for young adults and individual racial and ethnic minoritized subpopulations. Public health practitioners should consider subpopulation-specific messaging and programmatic strategies that address differences in MH needs, particularly for those historically marginalized.

## Key Points

**Question:**

How have adult mental health (MH)–related emergency department (ED) visits changed during the COVID-19 pandemic?

**Findings:**

In this cross-sectional study of 107 761 319 eligible ED visits, MH-related visit count findings depended on the COVID-19 pandemic period examined, whether this was compared with other periods in the pandemic or prepandemic period, and which mental disorder was examined. There was between- and within-group variation in ED visits by race and ethnicity, which varied by pandemic period examined, and there were increases in some disorders after COVID-19 case peaks for adults aged 18 to 24 years.

**Meaning:**

Results of this study suggest that EDs may have increases in MH-related visits after COVID-19 surges, especially for young adults and some racial and ethnic minoritized subpopulations.

## Teaser

This cross-sectional study investigates changes in adult mental health–related emergency department visits during the COVID-19 pandemic in the US.

## Introduction

The COVID-19 pandemic has resulted in greater self-reported symptoms of anxiety, depression, trauma and stress, tics, and other mental disorders among adults into early 2021.^1,2,3,4,5,6,7,8,9^ The pandemic may also have had an effect on mental health (MH)–seeking behavior. In addition to a rise in untreated mental disorders,^7^ there has been an increase in emergency department (ED) visits for selected aspects of MH in March to October 2020 compared with the prepandemic period.^10^ There are limited data on contemporary pandemic periods and insufficient information on MH care-seeking behavior and how this is affected by COVID-19 case surges, which is critical for health care planning.

Preexisting health, social, and economic conditions for historically marginalized communities in the US have exacerbated outcomes associated with the pandemic,^11,12,13,14^ including MH.^3,4,5^ Racial and ethnic minoritized groups, particularly Black and Hispanic persons, had more symptoms of some mental disorders compared with other groups early in the pandemic.^3,4,5^ The cumulative effect of high COVID-19 mortality, economic downturn, and trauma—including racially motivated violence—have MH consequences for racial and ethnic minoritized groups.^15,16,17,18^ EDs serve as a critical entry point for health care services for racial and ethnic minoritized groups owing to lack of primary care and medical home access.^19,20^ There are limited data on MH care-seeking behavior for these communities during the pandemic.

This analysis examined changes for all MH-related ED visits, and visits for 10 specific mental disorders, for adults aged 18 to 64 years during the pandemic. Data through August 2021 were used to describe MH trends into the Delta variant period on emergency MH care-seeking behavior for US adults, with an emphasis on identifying changes and racial and ethnic inequities in adult MH-related ED visits before and during COVID-19 case surges.

## Methods

### Participants and Procedures

In this epidemiologic cross-sectional study, we analyzed deidentified electronic medical record data on ED visits for adults aged 18 to 64 years collected via the National Syndromic Surveillance Program (NSSP) from January 1, 2019, to August 14, 2021. More than 3600 facilities from 49 states and District of Columbia contributed data at the time of analysis, constituting approximately 71% of US nonfederal ED facilities.^21^ Administrative data analyses are considered public health practice by the US Centers for Disease Control and Prevention; therefore, no institutional review board approval or patient consent was required. Race and ethnicity information for all adults was gathered from the database and included American Indian or Alaska Native, Asian, Black, Hispanic, Native Hawaiian or Other Pacific Islander, and White. Persons of Hispanic ethnicity were classified as Hispanic regardless of race; other categories reflect non-Hispanic persons. All MH-related ED visits, and visits related to 10 disorders (ie, anxiety, depressive, bipolar, schizophrenia spectrum, trauma- and stressor-related [TS], attention-deficit/hyperactivity, disruptive behavioral and impulse, obsessive-compulsive, eating, and tic disorders), were identified using chief complaint terms and discharge diagnosis codes (eTable 1 in the Supplement). This study followed the Strengthening the Reporting of Observational Studies in Epidemiology (STROBE) reporting guidelines.

### Statistical Analysis

As contributing facilities change over time and chief complaint and discharge diagnosis field completeness varies by facility, we limited all analyses to facilities reporting high-quality data (coefficient of variation ≤40; average weekly discharge diagnosis ≥75%) in each period of interest. For race and ethnicity analyses, we also restricted data to include only average weekly data at least 75% or more complete for combined race and ethnicity fields. As of August 2021, NSSP race and ethnicity data were 84.5% complete; completeness and data collection mechanisms vary by facility.

We examined MH-related ED visits before and during the COVID-19 pandemic; periods were selected by examining changes in US COVID-19 case trajectories.^22^ To examine the association of the Delta pandemic period with MH-related ED visits, we compared visits in a period with high (>90% of US specimens tested) Delta variant circulation (July 18-August 14, 2021) with a period when Delta circulation was low (<15%; April 18-May 15, 2021).^23^ We compared MH-related ED visits immediately after a COVID-19 case peak (February 14-March 13, 2021) with during a COVID-19 case peak (December 27, 2020-January 23, 2021) to understand trends in MH-related visits after COVID-19 case surges. We compared the Delta period and the period after a COVID-19 case peak with corresponding weeks in 2019 to understand changes in MH-related ED visits before and during the pandemic. Analytic index periods were either the COVID-19 Delta period (July 18-August 14, 2021) or the period after a COVID-19 case peak (February 14-March 13, 2021).

For each index period, raw ED visit counts and MH visits per 100 000 ED visits were calculated overall and by race and ethnicity, sex, and age. The denominator per 100 000 ED visits reflects visits for the subpopulation of interest in stratified results. To examine temporal changes from index to comparison periods, we calculated the percentage change in raw ED visit counts. In line with NSSP data standards, we considered changes in visit volume as follows: high decreases (≥−20%), moderate decreases (>−10% to −<20%), stable (≥−10% to <10%), moderate increases (≥10%-<20%), and high increases (≥20%). These thresholds included rounded percentages. In addition to presenting raw MH-related visit counts, we calculated visit ratios (VRs) and 95% CIs to compare the proportion of MH-related visits of all ED visits in the index with comparison periods. VRs and 95% CIs provided information on whether MH-related visits comprised a larger (VR >1.0) or smaller (VR <1.0) proportion of all ED visits in the index compared with comparison periods. The median (range) number of facilities included in analyses examining all MH-related ED visits and specific disorders overall and by sex and age was 2035 (1970-2041).

Owing to small cell sizes, analyses by race and ethnicity included only the 5 most common disorders and all MH-related visits. Owing to improvements in race and ethnicity data completeness during the pandemic, MH-related visit count percentage change and VRs with 95% CIs for race and ethnicity were only calculated when comparing the Delta with the pre-Delta period and the period after a COVID-19 case peak with during a peak. VRs with 95% CIs were calculated to compare the proportion of MH-related visits between racial and ethnic groups. There were 2352 facilities included in race and ethnicity analyses. All data were analyzed using R software, version 4.1.2 (The R Foundation).

## Results

This epidemiologic cross-sectional study included 107 761 319 ED visits among adults aged 18 to 64 years (16 569 474 [15%] aged 18-24 years; 58 682 874 [55%] aged 25-49 years; 32 508 971 [30%] aged 50-64 years; 59 870 475 [56%] women; 47 711 919 [44%] men) from January 1, 2019, to August 14, 2021. There were 2 510 744 ED visits included in the subsample used for the race and ethnicity analysis; sex and age demographic patterns mirrored those for the entire sample. Among these visits, 24 592 (1%) were among American Indian or Alaska Native persons, 33 697 (1%) were among Asian persons, 494 198 (20%) were among Black persons, 389 740 (16%) were among Hispanic persons, 5000 (0.2%) were among Native Hawaiian or Other Pacific Islander persons, and 1 172 683 (47%) were among White persons.

**Figure 1.**
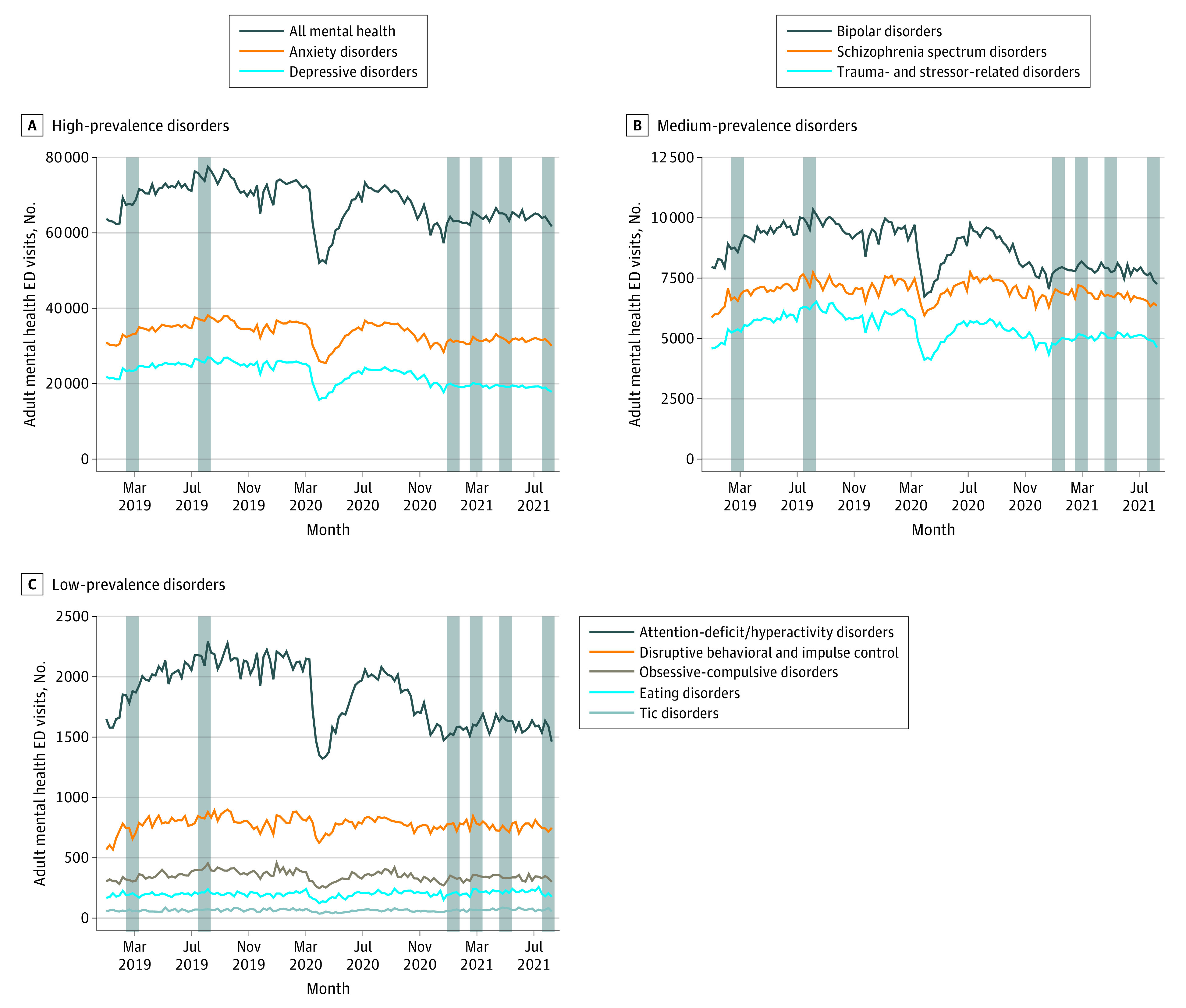
US Mental Health–Related Emergency Department (ED) Visit Counts for Adults Aged 18 to 64 years, Overall and by Disorder This figure includes data from January 1, 2019, through August 14, 2021. Shaded areas of the graph depict time periods included in the analytic index or comparison periods, which are as follows: the prepandemic comparison periods (February 10-March 9, 2019, and July 14-August 10, 2019), the COVID-19 case peak comparison period (December 27, 2020-January 23, 2021), the period immediately after a COVID-19 case peak index period (February 14-March 13, 2021), a COVID-19 pandemic comparison period with low circulation of the Delta variant, called the pre-Delta period (April 18-May 15, 2021), and a COVID-19 pandemic index period with high circulation of the Delta variant, called the Delta period (July 18-August 14, 2021). To accommodate differences in counts of all mental health–related visits and specific disorders, results for high-prevalence (A), medium-prevalence (B), and low-prevalence (C) disorders are presented.

MH-related ED visit counts from January 1, 2019, to August 14, 2021, are listed in Figure 1. After a decline in March 2020, MH-related ED visit counts for most disorders rose from the spring to the summer of 2020, with a decline in late fall 2020. MH-related ED visits, overall and for most disorders, were at their highest in the summer to midfall 2020.

### Delta to Pre-Delta Pandemic Period

Among adults aged 18 to 64 years, there was stability in all MH-related ED visit counts (−3.3%), as well as counts for most specific disorders (range, −1.4% to −7.5%), between the Delta and pre-Delta periods (Table 1). However, there were moderate and high decreases in ED visit counts for eating disorders (−11.9%) and tic disorders (−19.8%), respectively, from the Delta to the pre-Delta periods.

**Table 1.** Changes in MH-Related ED Visits for Adults Aged 18 to 64 Years in Selected Periods Before and During the COVID-19 Pandemic, US, February 10, 2019, to August 14, 2021^a^^,^^b^

Disorder	After Delta variant onset, index period: July 18-August 14, 2021						After a COVID-19 case peak, index period: February 14, 2021-March 13, 2021					
	Total ED visit counts for MH, No.	ED visits for MH per 100 000 ED visits^c^	Index period compared with MH in the COVID-19 pandemic before the Delta variant period (April 18-May 15, 2021)		Index period compared with prepandemic MH (July 14, 2019-August 10, 2019)		Total ED visit counts for MH, No.	ED visits for MH per 100 000 ED visits^c^	Index period compared with MH during a COVID-19 case peak (December 27, 2020-January 23, 2021)		Index period compared with prepandemic MH (February 10, 2019-March 9, 2019)	
			Change in ED counts for MH, %^d^	Visit ratio (95% CI)^e^	Change in ED counts for MH, %^d^	Visit ratio (95% CI)^e^			Change in ED counts for MH, %^d^	Visit ratio (95% CI)^e^	Change in ED counts for MH, %^d^	Visit ratio (95% CI)^e^
All MH	249 700	6725	−3.3	0.86 (0.85-0.86)	−17.2	0.80 (0.79-0.80)	256 628	8728	1.5	1.04 (1.03-1.04)	−5.4	1.11 (1.11-1.12)
Anxiety	122 463	3298	−3.4	0.86 (0.85-0.86)	−17.7	0.79 (0.78-0.80)	125 900	4282	0.6	1.03 (1.02-1.03)	−4.2	1.13 (1.12-1.14)
Depressive	72 674	1957	−5.7	0.84 (0.83-0.84)	−30.7	0.67 (0.66-0.67)	79 463	2703	1.2	1.03 (1.02-1.04)	−15.4	1.00 (0.99-1.01)
Bipolar	29 460	793	−6.2	0.83 (0.82-0.84)	−25.6	0.71 (0.70-0.73)	32 010	1089	2.2	1.04 (1.03-1.06)	−8.7	1.08 (1.06-1.09)
Schizophrenia spectrum	25 309	682	−6.4	0.83 (0.82-0.84)	−15.6	0.81 (0.80-0.83)	28 107	956	1.9	1.04 (1.02-1.06)	5.2	1.24 (1.22-1.26)
Trauma- and stressor-related	18 982	511	−6.3	0.83 (0.81-0.85)	−24.7	0.72 (0.71-0.74)	20 403	694	4.9	1.07 (1.05-1.09)	−3.9	1.13 (1.11-1.15)
Attention-deficit/ hyperactivity	6078	164	−7.5	0.82 (0.79-0.85)	−30.5	0.67 (0.65-0.69)	6337	216	3.4	1.06 (1.02-1.09)	−14.1	1.01 (0.98-1.05)
Disruptive behavioral and impulse	2898	78	−1.4	0.87 (0.83-0.92)	−14.2	0.82 (0.78-0.87)	3131	106	2.1	1.04 (0.99-1.10)	9.5	1.29 (1.23-1.36)
Obsessive-compulsive	1276	34	−5.8	0.83 (0.77-0.90)	−23.4	0.74 (0.68-0.79)	1373	47	3.8	1.06 (0.98-1.14)	9.8	1.29 (1.20-1.40)
Eating	761	21	−11.9	0.78 (0.71-0.86)	−12.1	0.84 (0.77-0.93)	869	30	6.9	1.09 (0.99-1.20)	11.1	1.31 (1.19-1.44)
Tic	263	7	−19.8	0.71 (0.60-0.84)	−6.4	0.90 (0.76-1.06)	276	9	7.4	1.10 (0.93-1.30)	11.3	1.31 (1.10-1.56)

Abbreviations: ED, emergency department; MH, mental health.

^a^ The National Syndromic Surveillance Program collects free-text reason for visit (chief complaint), discharge diagnosis, and patient demographic details. Diagnosis information is collected using *International Classification of Diseases, Ninth Revision, Clinical Modification* codes; *International Classification of Diseases, Tenth Revision, Clinical Modification* codes; and Systematized Nomenclature of Medicine codes. ED visits associated with any MH-related visit and specific mental disorders were identified by querying ED visits data from the National Syndromic Surveillance Program using keyword syndromes developed by the US Centers for Disease Control and Prevention in partnership with state and local health departments (eTable 1 in the Supplement).

^b^ To reduce artifactual effects from changes in reporting patterns, analyses examining trends in MH-related ED visits overall were restricted to facilities with a coefficient of variation of 40 or less and mean weekly discharge diagnosis of 75% or more from January 2019 to August 2021.

^c^ Rate of ED visits for MH outcome equaled the number of ED visits for MH outcome in the period of interest divided by the number of total ED visits in the period of interest multiplied by 100 000.

^d^ Percentage change in visits was calculated as the difference in total visits between the index period and the comparison period divided by the total visits during the comparison period multiplied by 100.

^e^ Visit ratios equaled the proportion of ED visits for MH outcome during the index period divided by the proportion of ED visits for MH outcome during the comparison period.

**Figure 2.**
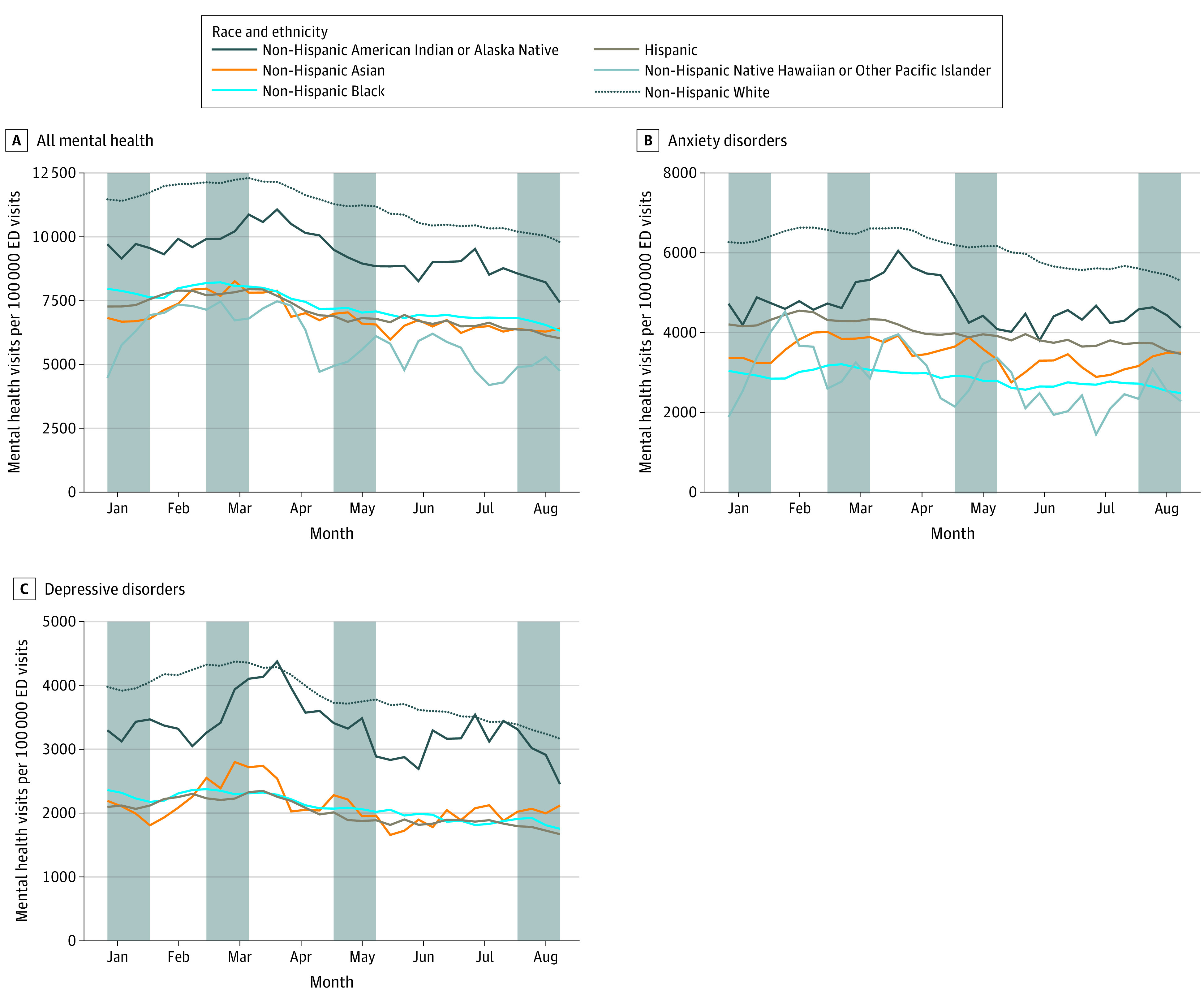
Mental Health–Related, Anxiety, and Depressive Disorder US Emergency Department (ED) Visits, by Race and Ethnicity, December 27, 2020, to August 14, 2021 This figure includes data for all mental health (A), anxiety disorders (B), and depressive disorders (C). Shaded areas of the graph depict time periods included in analytic index or comparison periods: a COVID-19 case peak comparison period (December 27, 2020-January 23, 2021), the period immediately after a COVID-19 case peak index period (February 14-March 13, 2021), a COVID-19 pandemic comparison period with low circulation of the Delta variant (the pre-Delta period; April 18-May 15, 2021), and a COVID-19 pandemic index period with high circulation of the Delta variant (the Delta period; July 18-August 14, 2021). These windows were used in the temporal comparisons displayed in Table 2 (eAppendix 1 in the Supplement).

**Figure 3.**
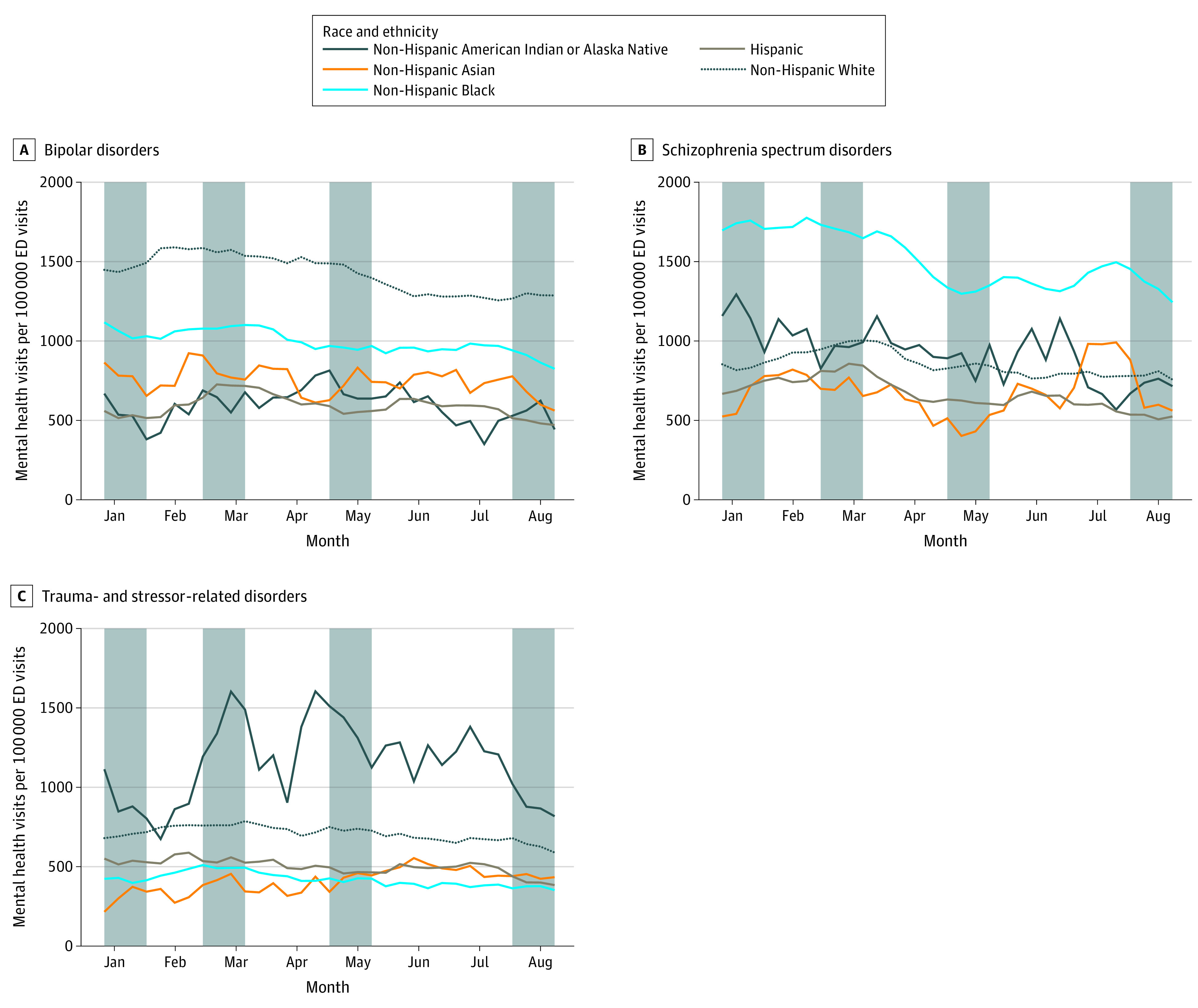
Bipolar, Schizophrenia Spectrum, and Trauma- and Stressor-Related Disorders US Emergency Department (ED) Visits, by Race and Ethnicity, December 27, 2020, to August 14, 2021 This figure includes data for bipolar disorders (A), schizophrenia spectrum disorders (B), and trauma- and stressor-related disorders (C). Shaded areas of the graph depict time periods included in analytic index or comparison periods: a COVID-19 case peak comparison period (December 27, 2020-January 23, 2021), the period immediately after a COVID-19 case peak index period (February 14-March 13, 2021), a COVID-19 pandemic comparison period with low circulation of the Delta variant (the pre-Delta period; April 18-May 15, 2021), and a COVID-19 pandemic index period with high circulation of the Delta variant (the Delta period; July 18-August 14, 2021). These windows were used in the temporal comparisons displayed in Table 2 (eAppendix 2 in the Supplement).

Across all MH-related visits and many disorders, White and American Indian or Alaska Native persons had a higher prevalence of ED visits for the disorder of interest per 100 000 ED visits than other groups (eg, all MH-related visits: American Indian or Alaska Native persons, 7428; Asian persons, 6406; Black persons, 6318; Hispanic persons, 6026; Native Hawaiian or Other Pacific Islander persons, 4744; White persons, 9792 per 100 000 ED visits) (Table 2, Figures 2 and 3, eAppendixes 1 and 2 in the Supplement). There were also differences between several other racial and ethnic minoritized groups in MH-related ED visits per 100 000 ED visits in the Delta period, which varied by specific disorders (Figures 2 and 3). For example, Black persons had a lower prevalence of anxiety-related ED visits (2481 per 100 000) than Asian persons (3497 per 100 000) and Hispanic persons (3462 per 100 000) (Figure 2B), but a higher prevalence of schizophrenia spectrum disorder-related ED visits (1244 per 100 000) compared with American Indian or Alaska Native (716 per 100 000), Asian (562 per 100 000), Hispanic (524 per 100 000), and White (757 per 100 000) persons (Figure 3B). MH-related ED visits varied over time within racial and ethnic groups (Table 2). Asian persons had a moderate increase (12.0%), and Native Hawaiian or Pacific Islanders had a moderate decrease (−16.0%) in all MH-related visit counts in the Delta period compared with the pre-Delta period; other groups had stable counts over these periods. Black, Hispanic, and White persons had stable ED visit counts for all specific disorders between the Delta and pre-Delta period. Although visit counts were stable for depressive disorders, American Indian or Alaska Native persons had a high decrease in ED visit counts for bipolar (−30.8%), schizophrenia spectrum (−23.8%), and TS-related (−25.0%) disorders—but a moderate increase in anxiety-related ED visits (9.9%)—in the Delta period compared with the pre-Delta period. Asian persons had moderate to high increases in ED visit counts for anxiety disorders (18.3%), depressive disorders (20.5%), schizophrenia spectrum disorders (18.2%), and TS-related disorders (11.8%); visits for bipolar disorders decreased (−13.6%).

**Table 2.** Changes in MH-Related ED Visits for Adults Aged 18 to 64 Years During Selected Periods During the COVID-19 Pandemic, by Race and Ethnicity, US, December 27, 2020, to August 14, 2021^a^^,^^b^

Race and ethnicity	After Delta variant onset, index period: July 18-August 14, 2021				After a COVID-19 case peak, index period: February 14, 2021-March 13, 2021			
	Total ED visit counts for MH, No.	ED visits for MH per 100 000 ED visits^c^	Index period compared with MH during the COVID-19 pandemic, prior to Delta variant period (April 18-May 15, 2021)		Total ED visit counts for MH, No.	ED visits for MH per 100 000 ED visits^c^	Index period compared with MH during a COVID-19 case peak (December 27, 2020-January 23, 2021)	
			Change in ED counts for MH, %^d^	Visit ratio (95% CI)^e^			Change in ED counts for MH, %^d^	Visit ratio (95% CI)^e^
**All MH**								
American Indian or Alaska Native	218	7428	−8.3	0.84 (0.70-1.00)	241	10 871	6.2	1.14 (0.96-1.35)
Asian	251	6406	12.0	0.98 (0.82-1.16)	227	7809	4.0	1.15 (0.96-1.37)
Black	3693	6318	1.7	0.89 (0.85-0.93)	3489	8050	3.0	1.06 (1.01-1.10)
Hispanic	2792	6026	−0.5	0.89 (0.84-0.93)	2783	7946	−0.3	1.05 (1.00-1.11)
Native Hawaiian or Other Pacific Islander	25	4744	−16.0	0.78 (0.46-1.31)	31	6798	0.0	0.98 (0.61-1.58)
White	13 226	9792	2.2	0.87 (0.86-0.90)	12 388	12 301	2.0	1.05 (1.02-1.07)
**Anxiety disorders**								
American Indian or Alaska Native	121	4123	9.9	1.01 (0.78-1.30)	118	5323	5.1	1.12 (0.87-1.45)
Asian	137	3497	18.3	1.05 (0.82-1.34)	113	3887	8.0	1.20 (0.92-1.56)
Black	1450	2481	1.3	0.89 (0.83-0.96)	1328	3064	5.0	1.08 (1.00-1.16)
Hispanic	1604	3462	−0.9	0.88 (0.83-0.95)	1518	4334	−5.1	1.00 (0.94-1.07)
Native Hawaiian or Other Pacific Islander	12	2277	−33.3	0.68 (0.32-1.41)	13	2851	−38.5	0.71 (0.35-1.43)
White	7157	5299	0.4	0.86 (0.83-0.89)	6655	6608	0.1	1.03 (1.00-1.06)
**Depressive disorders**								
American Indian or Alaska Native	72	2453	−6.9	0.85 (0.62-1.17)	91	4105	9.9	1.18 (0.88-1.59)
Asian	83	2118	20.5	1.08 (0.79-1.49)	79	2718	26.6	1.50 (1.08-2.10)
Black	1025	1754	−1.1	0.87 (0.80-0.95)	1001	2310	3.6	1.06 (0.97-1.16)
Hispanic	773	1668	−0.9	0.88 (0.80-0.98)	815	2327	4.1	1.10 (1.00-1.21)
Native Hawaiian or Other Pacific Islander	NA^f^	NA^f^	NA^f^	NA^f^	NA^f^	NA^f^	NA^f^	NA^f^
White	4273	3164	−2.2	0.84 (0.80-0.87)	4386	4355	4.3	1.07 (1.03-1.12)
**Bipolar disorders**								
American Indian or Alaska Native	13	443	−30.8	0.70 (0.34-1.43)	15	677	40.0	1.78 (0.78-4.05)
Asian	22	562	−13.6	0.76 (0.43-1.34)	22	757	4.6	1.16 (0.64-2.10)
Black	482	825	−3.1	0.85 (0.75-0.96)	477	1101	4.2	1.07 (0.94-1.21)
Hispanic	218	471	−6.0	0.84 (0.70-1.01)	251	717	24.3	1.39 (1.15-1.68)
Native Hawaiian or Other Pacific Islander	NA^f^	NA^f^	NA^f^	NA^f^	NA^f^	NA^f^	NA^f^	NA^f^
White	1738	1287	7.1	0.92 (0.86-0.99)	1547	1536	0.1	1.03 (0.96-1.10)
**Schizophrenia spectrum disorders**								
American Indian or Alaska Native	21	716	−23.8	0.73 (0.41-1.30)	22	992	0	1.07 (0.59-1.92)
Asian	22	562	18.2	1.05 (0.56-1.96)	19	654	−31.6	0.84 (0.46-1.52)
Black	727	1244	4.7	0.92 (0.83-1.02)	714	1647	−6.0	0.97 (0.87-1.07)
Hispanic	243	524	−2.9	0.87 (0.73-1.03)	296	845	6.4	1.13 (0.96-1.33)
Native Hawaiian or Other Pacific Islander	NA^f^	NA^f^	NA^f^	NA^f^	NA^f^	NA^f^	NA^f^	NA^f^
White	1022	757	4.5	0.90 (0.82-0.98)	1011	1004	11.5	1.16 (1.06-1.27)
**Trauma- and stressor-related disorders**								
American Indian or Alaska Native	24	818	−25.0	0.73 (0.43-1.24)	33	1488	42.4	1.85 (1.06-3.25)
Asian	17	434	11.8	0.97 (0.49-1.95)	10	344	−10.0	1.00 (0.43-2.36)
Black	206	352	−5.8	0.83 (0.69-1.00)	214	494	14.0	1.19 (0.98-1.45)
Hispanic	178	384	−7.9	0.83 (0.68-1.01)	184	525	−6.0	0.99 (0.81-1.22)
Native Hawaiian or Other Pacific Islander	NA^f^	NA^f^	NA^f^	NA^f^	NA^f^	NA^f^	NA^f^	NA^f^
White	796	589	−5.5	0.81 (0.74-0.89)	792	786	6.2	1.10 (0.99-1.21)

Abbreviations: ED, emergency department; MH, mental health; NA, not available.

^a^ The National Syndromic Surveillance Program collects free-text reason for visit (chief complaint), discharge diagnosis, and patient demographic details. Diagnosis information is collected using *International Classification of Diseases, Ninth Revision, Clinical Modification* codes; *International Classification of Diseases, Tenth Revision, Clinical Modification* codes; and Systematized Nomenclature of Medicine codes. ED visits associated with any MH-related visit and specific mental disorders were identified by querying ED visits data from the National Syndromic Surveillance Program using keyword syndromes developed by the US Centers for Disease Control and Prevention in partnership with state and local health departments (eTable 1 in the Supplement).

^b^ To reduce artifactual effects from changes in reporting patterns, analyses examining trends in MH-related ED visits by race and ethnicity were restricted to facilities with a coefficient of variation of 40 or less and mean weekly discharge diagnosis of 75% or more from January to August 2021 and were further limited to facilities with 75% or more complete data for the combined race and ethnicity fields.

^c^ Rate of ED visits for MH outcome equaled the number of ED visits for MH outcome in the period of interest divided by the number of total ED visits in the time period of interest for the subpopulation of interest multiplied by 100 000.

^d^ Percentage change in visits was calculated as the difference in total visits between the index period and the comparison period divided by the total visits during the comparison period multiplied by 100.

^e^ Visit ratios equaled the proportion ED visits for MH outcome during the index period divided by the proportion of ED visits for MH outcome during the comparison period.

^f^ If index period ED visits counts for a particular race and ethnicity were less than 10, cell volumes were suppressed and are listed as NA.

VRs with 95% CIs, including for some racial and ethnic groups, were mostly less than 1.0 (Tables 1 and 2), indicating MH-related ED visits composed a smaller proportion of all ED visits in the Delta period compared with the pre-Delta period. For American Indian or Alaska Native and Asian persons, VRs and 95% CIs mostly spanned 1.0, indicating no difference in the proportion of MH-related ED visits in the Delta period compared with the pre-Delta period. Findings by sex and for those aged 25 to 49 years and 50 to 64 years mostly reflected nonstratified findings (eTable 2 in the Supplement). For adults aged 18 to 24 years, many disorders had moderate to high decreases in ED visit counts in the Delta period compared with the pre-Delta period.

### Changes Immediately After a COVID-19 Case Peak

Among adults aged 18 to 64 years, there was stability in adult MH-related ED visit counts overall (1.5%), including for specific disorders (range, 0.6%-7.4%) after a COVID-19 case peak compared with during a peak (Table 1). Similar to the Delta period findings, there were between-group racial and ethnic differences in ED visit prevalence for all MH-related visits and specific disorders (Figures 2 and 3).

For within-group differences, there was stability in all MH-related ED visit counts during and immediately after a COVID-19 case peak for all specific racial and ethnic subpopulations (range, −0.3% to 6.2%) (Table 2). For Black, Hispanic, and White persons, there was limited variability in ED visit counts for most disorders during this period. However, each of these groups had increases in the percentage change in visit counts for 1 disorder in the period after a COVID-19 case peak compared with during a peak (Black persons, 14.0% increase in TS-related disorder visits; Hispanic persons, 24.3% increase in bipolar disorder visits; White persons, 11.5% increase in schizophrenia spectrum disorder visits) in the period after a COVID-19 case peak. For Asian persons, there was stability in ED visit counts for several disorders, but high increases in depressive disorders (26.6%) and decreases in schizophrenia spectrum disorders (−31.6%) in the period after a COVID-19 case peak. Among American Indian or Alaska Native persons, anxiety and schizophrenia spectrum–related ED visit counts were stable after a COVID-19 case peak, but there were moderate to high increases in ED visit counts for other disorders (depressive disorders, 9.9%; bipolar disorders, 40.0%; TS-related disorders, 42.4%).

For MH-related visits overall and specific disorders, VRs were mostly greater than 1.0 with 95% CIs excluding 1.0 (Tables 1 and 2). This predominantly indicates that MH-related ED visits composed a larger proportion of all ED visits in the period immediately after a COVID-19 case peak compared with during a peak. For some racial and ethnic groups, VR point estimates were similar but with wider CIs that often spanned 1.0, which could be due to small sample size. Findings by age and sex reflected overall patterns, with several exceptions (eTable 2 in the Supplement): there was a moderate increase (10.9%) in eating disorder–related ED visit counts for men; high increases (25.8%) in tic disorder–related ED visit counts for women; and moderate to high increases in visit counts for 3 disorders (disruptive behavioral and impulse disorders, 11.8%; eating disorders, 12.5%, tic disorders, 25.8%) among adults aged 18 to 24 years after a COVID-19 case peak compared with during a peak.

### MH-Related ED Visits Compared With Prepandemic

Among adults aged 18 to 64 years, there were decreases for all MH-related ED visit counts (−17.2%) and for most disorders (range, −12.1% to −30.7%) when comparing the Delta period with the corresponding period in 2019, except for tic disorders, which was stable (−6.4%) (Table 1). Across most disorders and all MH-related visits, VRs were mostly less than 1.0 with 95% CIs that excluded 1.0 (Table 1), indicating that MH-related ED visits were a smaller proportion of ED visits in the Delta period compared with the corresponding prepandemic period in 2019. Findings by sex and age mostly reflected nonstratified results (eTable 2 in the Supplement).

For adults aged 18 to 64 years, ED visit counts for all MH-related visits (−5.4%) and for anxiety (−4.2%), bipolar (−8.7%), schizophrenia (5.2%), and TS (−3.9%) disorders were stable when comparing the period after a COVID-19 case peak with the corresponding prepandemic period (Table 1). There were moderate increases in ED visit counts for disruptive behavioral and impulse disorders (9.5%), obsessive-compulsive disorders (9.8%), eating disorders (11.1%), and tic disorders (11.3%), and moderate decreases for 2 disorders (attention-deficit/hyperactivity and depressive disorders), when comparing the period after a COVID-19 case peak with the corresponding prepandemic period. When stratified by sex and age, findings were similar for the most common disorders. However, increases in ED visit counts for obsessive-compulsive disorder were greater for men (18.8%) and particularly for those aged 50 to 64 years (19.2%). For eating and tic disorders, women (eating, 12.7%; tic, 34.1%) and people aged 18 to 24 years (eating, 22.8%; tic, 73.3%) had the greatest increases (eTable 2 in the Supplement). VRs were mostly greater than 1.0 with 95% CIs that excluded 1.0 (Table 1), indicating that MH-related ED visits composed a larger proportion of all ED visits after a COVID-19 case peak compared with the corresponding prepandemic period.

## Discussion

The COVID-19 pandemic has had collateral effects on MH^1,2,3,4,5,6,7,8,9^; to our knowledge, this epidemiologic cross-sectional study was the first analysis to examine differences in MH-related ED visits in the Delta period and to investigate the association of COVID-19 case surges with MH-related ED visits. Study findings suggest that all MH-related ED visit counts were mostly stable into the Delta period (except among Asian persons who had visit increases and people of Native Hawaiian or Other Pacific Islander race and ethnicity who had visit decreases) compared with a pre-Delta period but had declined since the corresponding prepandemic period. MH-related ED visits compose a smaller proportion of all ED visits in this period compared with earlier in the pandemic and prepandemic period. Given that self-reported MH concerns have increased during the pandemic,^1,2,3,4,5,6,7,8,9^ poor MH may be untreated or undertreated. Although it is possible that increases in primary care telemedicine use^24,25,26^ or other services may be offsetting some decreases in MH-related ED visits during these periods, these resources may be less available to disproportionately affected subpopulations^27^—such as racial and ethnic minoritized groups—who have historically been more likely to use EDs as first-line health care.^19,20^ Given the effect poor MH can have on life-long health,^28^ access to flexible MH services tailored to individual needs and accounting for differences in service access and availability are critical.

Study findings suggest that MH-related ED visit counts were mostly stable when comparing the period after a COVID-19 case peak with during a peak; some exceptions included adults aged 18 to 24 years. Compared with the prepandemic period, there were increases in ED visit counts for 4 disorders, decreases for 2 disorders, and stability for the remaining disorders and all MH-related visits. When accounting for ED visit volume,^29,30^ our results indicated that MH-related visits composed a larger proportion of ED visits after COVID-19 peaks compared with the corresponding prepandemic period. This aligns with previous work examining MH-related ED visits in 2020, which indicated that MH-related visits accounted for a higher proportion of visits than during the prepandemic period.^10^ Increases in MH-related ED visit counts after a COVID-19 case peak were notable for several racial and ethnic minoritized groups, including American Indian or Alaska Native persons. The increases observed after a COVID-19 case peak may suggest that individuals are experiencing stress and increases in poor MH in the periods after a COVID-19 case surge, as witnessed after a public health crisis.^31,32,33^ Alternatively, individuals may be delaying MH care during COVID-19 peaks owing to limited accessibility or perceived safety of services,^34^ or care may be unavailable given reduced capacity. After a COVID-19 case peak, hospitals may need to prepare for a potential increase in MH care in emergency health care settings.

Study results showed variation in patterns for MH care-seeking, which change based on the time of the pandemic, both in ED visits within and across racial and ethnic groups. This highlights the need to support persons who have used EDs as a safety net during the prepandemic period.^19,20^ The variation in MH-related ED visits, including across specific disorders, by racial and ethnic group subpopulation suggests that communication and advocacy interventions may need to be tailored for groups who are experiencing increases in poor MH during the pandemic. Within-group variability of MH-related ED visits during the pandemic suggests the need for attention from clinicians related to continuity of care for those with preexisting MH problems. Focus on proactive and preventive care for racial and ethnic groups more likely to experience new MH needs during the pandemic may be warranted. Race and ethnicity are related to inequities in hospital care and mortality, and stigmatizing experiences and other factors that lead to mistrust.^35,36^ Primary prevention efforts are needed to maintain mental and physical health, sustain community resilience, and overcome additive stress owing to the COVID-19 pandemic.

### Limitations

This study had some limitations. Although NSSP data represent approximately 71% of US ED facilities, these data are not nationally representative. After limiting our study to facilities that reported high-quality data, results may not be generalizable to all NSSP-contributing facilities. ED visit counts should be considered an underestimate of MH-related disorders that are identified in ED visit records, given that these data do not include all US EDs. There were substantial changes in ED health care–seeking behavior during the pandemic,^29,30^ which may have affected results as the characteristics of persons seeking care before and during the pandemic may have changed. Although all examined MH definitions were validated on NSSP data from multiple US jurisdictions, there may have been misclassification owing to challenges with identifying and reporting MH-related visits. We could not distinguish between acute and chronic presentation of MH-related visits, or visits where MH was a comorbidity but not the primary reason for the visit. Estimates provided do not capture all health care–seeking needs for persons experiencing MH challenges given that many people receive MH care outside of EDs. Moreover, this analysis focused on MH and did not include suicide or other behavioral health–related outcomes, which are critical and should be examined in future research. Finally, there may have been misclassification in race and ethnicity, which could influence estimates of inequities in MH-related ED visit burden.

## Conclusions

This epidemiologic cross-sectional study described changes in MH-related ED visits into the Delta variant pandemic period and during and after peaks in US COVID-19 cases. The findings suggest that there were fluctuations in patterns of MH-related ED visits relative to COVID-19 case surges, which differed by race and ethnicity. Emergency health care professionals may experience increases in MH-related visits during COVID-19 case surges (such as during the Delta period) among Asian persons and after COVID-19 case surges, particularly for adults aged 18 to 24 years and for American Indian or Alaska Native persons. There may also be increases in visits for specific disorders for individual racial and ethnic groups. Public health practitioners should consider subpopulation-specific messaging and programmatic strategies that address differences in MH prevention and intervention needs across subpopulations, particularly those who have been marginalized in the US.

## Notes

Supplement. **eTable 1. **Chief Complaint Keywords and Discharge Diagnosis Codes Included in Definitions for Emergency Department Visits Related to Mental Health **eTable 2. **Changes in Mental Health–Related Emergency Department Visits for Adults Aged 18 to 64 Years in Selected Periods Before and During the COVID-19 Pandemic, by Sex and Age, US, February 10, 2019, to August 14, 2021 **eAppendix 1. **Detailed Footnotes for Figure 2 Mental Health–Related, Anxiety, and Depressive Disorder US Emergency Department Visits, by Race and Ethnicity **eAppendix 2. **Detailed Footnotes for Figure 3 Bipolar, Schizophrenia Spectrum, and Trauma- and Stressor-Related Disorder US Emergency Department Visits, by Race and Ethnicity
