# Supplementary material for: Changes and Inequities in Adult Mental Health–Related Emergency Department Visits During the COVID-19 Pandemic in the US
Source: JAMA Psychiatry. 2022 Mar 16;79(5):475–85. doi: 10.1001/jamapsychiatry.2022.0164 (PMC8928092; doi:10.1001/jamapsychiatry.2022.0164)
Supplement: Supplement. — eTable 1. Chief Complaint Keywords and Discharge Diagnosis Codes Included in Definitions for Emergency Department Visits Related to Mental Health eTable 2. Changes in Mental Health–Related Emergency Department Visits for Adults Aged 18 to 64 Years in Selected Periods Before and During the COVID-19 Pandemic, by Sex and Age, US, February 10, 2019, to August 14, 2021 eAppendix 1. Detailed Footnotes for Figure 2 Mental Health–Related, Anxiety, and Depressive Disorder US Emergency Department Visits, by Race and Ethnicity eAppendix 2. Detailed Footnotes for Figure 3 Bipolar, Schizophrenia Spectrum, and Trauma- and Stressor-Related Disorder US Emergency Department Visits, by Race and Ethnicity [file jamapsychiatry-e220164-s001.pdf]

# Supplementary Online Content

Anderson KN, Radhakrishnan L, Lane RI, et al. Changes and inequities in adult mental health–related emergency department visits during the COVID-19 pandemic in the US. *JAMA Psychiatry.* Published online March 16, 2022. doi:10.1001/jamapsychiatry.2022.0164

**eTable 1.** Chief Complaint Keywords and Discharge Diagnosis Codes Included in Definitions for Emergency Department Visits Related to Mental Health

**eTable 2.** Changes in Mental Health–Related Emergency Department Visits for Adults Aged 18 to 64 Years in Selected Periods Before and During the COVID-19 Pandemic, by Sex and Age, US, February 10, 2019, to August 14, 2021

**eAppendix 1.** Detailed Footnotes for Figure 2 Mental Health–Related, Anxiety, and Depressive Disorder US Emergency Department Visits, by Race and Ethnicity

**eAppendix 2.** Detailed Footnotes for Figure 3 Bipolar, Schizophrenia Spectrum, and Trauma- and Stressor-Related Disorder US Emergency Department Visits, by Race and Ethnicity

This supplementary material has been provided by the authors to give readers additional information about their work.

© 2022 Anderson KN et al. *JAMA Psychiatry.*

**eTable 1.** Chief Complaint Keywords and Discharge Diagnosis Codes Included in Definitions for Emergency Department Visits Related to Mental Health

**Description:**
The purpose of these syndromes is to identify visits among persons who experienced any mental health challenge, including specific disorders, in emergency departments and ambulatory healthcare settings. This includes capturing visits where there are acute mental health crises (i.e., the sole or primary reason for the visit is only related to mental health) as well as visits where mental disorders are present (defined as coded in the discharge diagnosis or mentioned in the chief complaint text) but may not be the sole reason for the visit.

This definition uses *International Classification of Diseases, Ninth Revision, Clinical Modification* codes (ICD-9-CM), *International Classification of Diseases, Tenth Revision, Clinical Modification* codes (ICD-10-CM), Systematized Nomenclature of Medicine (SNOMED) codes, and free text reason for visit (chief complaint terms).

-- Not shown: Non-decimal versions of all discharge diagnosis codes were included.
-- Not shown: Common misspellings of chief complaint terms were included.

| Definition | Chief Complaint Search Terms | Diagnosis Codes |
|---|---|---|
| **CDC Mental Health v1** | *Also includes chief complaint terms from the conditions listed below*<br><br>Grief (include misspellings)<br>Grieve (include misspellings)<br>Bereave (include misspellings)<br>Psych<br>Mental health<br>Mental evaluation<br>Mental problem<br>Mentally<br>Mental illness<br>Reactive attachment<br>Auditory hallucination<br>Visual hallucination<br>Hearing voice<br>Evaluation hallucination<br>History of hallucination<br>Hallucinations patient<br>Hallucinations unspecified<br>Seeing thing<br>Delusion (include misspellings)<br>Paranoia (include misspellings)<br>Trichotillomania<br>Dissociative disorder<br>Dissociative conversion<br>Dissociative identity<br>Dissociative and conversion<br>Dissociative tendency<br>Dissociative episode<br>Dissociative conversion<br>Dissociative behavior<br>Dissociative amnesia<br>Dissociative attentive<br>Experiencing dissociation<br>Dissociative (when used with depress, | *Also includes ICD-10-CM, ICD-9-CM, SNOMED codes from the conditions listed below*<br><br>296.90; 296.99; 297.0; 297.1; 297.2; 297.3; 297.8; 297.9; 298.0; 298.1; 298.2; 298.3; 298.4; 298.8; 298.9; 300.10; 300.11; 300.12; 300.13; 399.14; 300.15; 300.81; 300.82; 300.89; 300.7; 307.80; 307.89; 300.9<br>648.40, 648.41, 648.42, 648.43,648.44<br>V62.82<br><br>F22; F23; F28; F29; F39; F44; F45; F48; F53.1; F54; F63.3; F93.8; F93.9; F94; F98.9<br>F99; Z63.4<br><br>413307004; 74732009; 231538003; 268664001; 48500005; 191667009; 41526007; 18193002; 224965009;<br>74506000 |

© 2022 Anderson KN et al. *JAMA Psychiatry.*

| | | |
|---|---|---|
| | depression, psych, dysthymic, anxiety, anxious, traumatic stress, PTSD, adjustment disorder, mental, attention deficit, bipolar, schizophrenia, schizoaffective, schizotypal – and associated misspellings of all terms) | |
| **CDC Anxiety Disorders v1** | Anxiety (include misspellings)<br>Anxious<br>Panic<br>Phobia | 300.00; 300.01; 300.02; 300.09; 300.20; 300.21; 300.22; 300.23; 300.29; 313.0<br>309.24; 309.28<br><br>F40; F41; F43.22; F43.23; F93.0<br><br>197480006; 126943008; 21897009; 69479009; 109006; 61387006; 70997004; 191709001 |
| **CDC Depressive Disorders v1** | Depression<br>Dysthymic<br>Dysthymia<br>Mood disorder<br>Mood disturbance | 296.20; 296.21; 296.22; 296.23; 296.24; 296.25; 296.30; 296.31; 296.32; 296.33; 296.34; 296.35; 300.4; 309.0; 309.1; 296.90; 296.99; 309.28<br><br>F32 (except F32.5); F33 (except F33.42); F34.1; F34.9; F43.21; F43.23; O90.6<br><br>35489007; 310497006; 370143000; 712823008; 57194009 |
| **CDC Bipolar Disorders v1** | Bipolar<br>Manic<br>Cyclothymic<br>Mania<br>Hypomania<br>Hypermania | 296.00; 296.01; 296.02; 296.03; 296.04; 296.05; 296.40; 296.41; 296.42; 296.43; 296.44; 296.45; 296.50; 296.51; 296.52; 296.53; 296.54; 296.55; 296.60; 296.61; 296.62; 296.63; 296.64; 296.65; 296.7<br>296.80; 296.81; 296.82; 296.89<br><br>F30.1; F30.2; F30.3; F30.8; F30.9; F31.0; F31.1; F31.2; F31.3; F31.4; F31.5; F31.6; F31.7 (only include: F31.70, F31.71, F31.73, F31.75, F31.77); F31.8; F31.9; F34.0<br><br>191627008; 371596008; 83225003;<br>231494001; 284513006; 268619003 |
| **CDC Schizophrenia Spectrum Disorders v1** | Schizophrenia (include misspellings)<br>Schizotypal (include misspellings)<br>Schizoaffective (include misspellings) | 295.00; 295.01; 295.02; 295.03; 295.04; 295.10; 295.11; 295.12; 295.13; 295.14; 295.20; 295.21; 295.22; 295.23; 295.24; 295.30; 295.31; 295.32; 295.33; 295.34; 295.40; 295.41; 295.42; 295.43; |

© 2022 Anderson KN et al. *JAMA Psychiatry.*

| | | |
|---|---|---|
| | | 295.44; 295.50; 295.51; 295.52; 295.53; 295.54; 295.60; 295.61; 295.62; 295.63; 295.64; 295.70; 295.71; 295.72; 295.73; 295.74; 295.80; 295.81; 295.82; 295.83; 295.84; 295.90; 295.91; 295.92; 295.93; 295.94<br><br>F20; F21; F25<br><br>58214004; 268617001; 64905009; 191526005; 68890003 |
| **CDC Trauma and Stressor-related Disorders v1** | Traumatic stress<br>PTSD (including misspellings)<br>Adjustment disorder<br>Reaction to acute stress<br>Acute stress reaction<br>Acute stress crisis<br>Acute stress eval<br>Eval acute stress<br>Acute stress behavior (including misspellings for behavior)<br>Behavior acute stress<br>Behavior acute stress<br>Reaction to severe stress<br>Severe stress reaction<br>Severe stress crisis | 308.0; 308.1; 308.2; 308.3; 308.4; 308.9; 309.81; 309.89; 309.9; 309.0; 309.1; 309.24; 309.28;309.29; 309.3; 309.4<br><br>F43<br><br>67195008; 47505003; 17226007; 57194009;<br>271952001 |
| **CDC Attention-Deficit/Hyperactivity Disorders v1** | Attention deficit<br>Hyperactivity<br>Attention problems | 314.00, 314.01; 314.9; 314.2<br><br>F90 (include all in this series)<br><br>406506008<br>35253001 |
| **CDC Disruptive Behavioral and Impulse-Control v1** | Conduct disorder<br>Oppositional defiant (with spelling mistakes for “defiant”)<br>Antisocial personality<br>Disturbance of conduct<br>Impulse control disorder<br>Explosive disorder | 309.3; 309.4; 312.00; 312.01; 312.02; 312.03; 312.10; 312.11; 312.12; 312.13; 312.20, 312.21; 312.22; 312.23; 312.4; 312.81; 312.82; 312.89; 312.9; 312.30; 312.31; 312.39; 313.81; 314.2; 301.7<br><br>F43.24; F43.25; F91.0; F91.1; F91.2; F91.3; F91.8; F91.9; F63.81;<br>F63.1; F63.2; F63.9; F63.0; F63.89; F60.2<br><br>54319003; 18941000; 430909002; 26665006 |
| **CDC Obsessive-Compulsive Disorders v1** | Obsessive compulsive<br>Compulsive disorder | 300.3<br><br>F42<br><br>191736004 |

© 2022 Anderson KN et al. *JAMA Psychiatry.*

| | | |
|---|---|---|
| **CDC Eating Disorders v1** | Pica<br>Bulimia (including misspellings)<br>Binge eating (including misspellings of binge)<br>Binge-eating<br>Eating disorder<br>Rumination disorder<br>Binging or purging<br>Binging and purging<br>Weight loss or anorexia<br>Anorexia nervosa (including misspellings of anorexia)<br>Anorexic (including misspellings of anorexic)<br>Being treated for anorexia (including misspellings of anorexia)<br>Treatment for (including misspellings of anorexia)<br>Weight loss (including misspellings of weight; include when used with anorexia)<br>Loss of weight (including misspellings of weight; include when used with anorexia)<br>Weight loss (including misspellings of weight; include when used with anorexia)<br>Loss of weight (including misspellings of weight; include when used with anorexia)<br>Eating disorder<br>Anorexia history (including misspellings of anorexia)<br>History of anorexia (including misspellings of anorexia)<br>History anorexia (including misspellings of anorexia)<br>Mental anorexia (including misspellings of anorexia)<br>Mental (when used with anorexia)<br>Psych (when used with anorexia) | 307.1; 307.50; 307.51; 307.52; 307.53; 307.54; 307.59<br><br>F50.00; F50.01; F50.02; F50.2; F50.8; F50.82; F50.89; F50.9; F98.21; F98.29;<br>F98.3<br><br>72366004; 56882008; 7800400; 32721004 |
| **CDC Tic Disorders v1** | Neurological tic<br>Tic disorder<br>Behavioral tic<br>Facial tic<br>Face tic<br>Tic like phenomenon<br>Verbal tic<br>Motor tic | 307.20; 307.21; 307.22; 307.23<br><br>F95; G25.69<br><br>568005; 5158005 |

*Footnotes.* In developing the syndrome definitions noted above for mental health, a team of scientists with cross-cutting expertise in mental disorders – including a practicing psychiatrist – reviewed all ICD-9, ICD-10 and SNOMED codes related to mental disorders. Codes related to mental disorders that are non-specific to mental health, for example, related to substance use, developmental disabilities, or dementia, were excluded. Mental health-related codes were further classified into the aforementioned categories based on previous literature and with expert judgement based on best alignment with the *Diagnostic and Statistical Manual of Mental Disorders, version 5 (DSM-5)*. For individual disorder categories where the sample size in national syndromic surveillance data was too minimal to meaningfully include an individual syndrome definition, codes were placed in the "All Mental Health" definition (e.g., reactive attachment disorder). Terms and codes that were non-specific and unable to differentiate between specific

© 2022 Anderson KN et al. *JAMA Psychiatry.*

mental disorders (e.g., psychosis; hallucinations) were also included in the “All Mental Health” but not specific individual disorder syndromes to improve syndrome specificity.

© 2022 Anderson KN et al. *JAMA Psychiatry.*

**eTable 2**. Changes in Mental Health–Related Emergency Department Visits for Adults Aged 18 to 64 Years in Selected Periods Before and During the COVID-19 Pandemic, by Sex and Age, US, February 10, 2019, to August 14, 2021

| | | **Mental Health during the COVID-19 Pandemic, after Delta variant onset** Index Period: July 18–Aug. 14, 2021 | | | | | | **Mental Health during the COVID-19 Pandemic, following a COVID-19 case peak** Index Period: Feb. 14, 2021–Mar. 13, 2021 | | | | | |
|---|---|---|---|---|---|---|---|---|---|---|---|---|---|
| | | | | **Index period compared to MH in the COVID-19 Pandemic, prior to Delta variant period (Apr. 18–May 15, 2021)** | | **Index period compared to pre-pandemic MH (July 14, 2019–Aug. 10, 2019)** | | | | **Index period compared to MH during a COVID-19 case peak (Dec. 27, 2020–Jan. 23, 2021)** | | **Index period compared to pre-pandemic MH (Feb. 10, 2019–Mar. 9, 2019)** | |
| | | **Total ED visit counts for MH** | **ED visits for MH per 100,000 ED visits[c]** | **% Change in ED counts for MH[d]** | **Visit ratio (95% CI)[e]** | **% Change in ED counts for MH[d]** | **Visit ratio (95% CI)[e]** | **Total ED visit counts for MH** | **ED visits for MH per 100,000 ED visits[c]** | **% Change in ED counts for MH[d]** | **Visit ratio (95% CI)[e]** | **% Change in ED counts for MH[d]** | **Visit ratio (95% CI)[e]** |
| **All mental health** | **Sex** | | | | | | | | | | | | |
| | Females | 135,357 | 6615 | -3.0% | 0.86 (0.86, 0.87) | -20.2% | 0.78 (0.77, 0.78) | 138,098 | 8527 | 1.5% | 1.04 (1.03, 1.04) | -9.0% | 1.11 (1.10, 1.12) |
| | Males | 113,676 | 6848 | -3.6% | 0.85 (0.84, 0.85) | -13.5% | 0.82 (0.81, 0.83) | 117,899 | 8957 | 1.3% | 1.04 (1.03, 1.04) | -0.9% | 1.11 (1.10, 1.12) |
| | Male/Female Ratio[f] | | | | 1.04 (1.03, 1.04) | | 1.04 (1.03, 1.04) | | | | 1.05 (1.04, 1.06) | | 1.05 (1.04, 1.06) |
| | **Age** | | | | | | | | | | | | |
| | Aged 18-24 | 36,365 | 6401 | -6.7% | 0.83 (0.82, 0.84) | -12.6% | 0.85 (0.84, 0.87) | 37,945 | 8741 | 6.0% | 1.07 (1.06, 1.09) | -3.0% | 1.21 (1.19, 1.22) |
| | Aged 25-49 | 142,927 | 7012 | -2.4% | 0.86 (0.85, 0.86) | -15.9% | 0.81 (0.80, 0.81) | 144,811 | 9085 | 0.6% | 1.03 (1.03, 1.04) | -4.4% | 1.13 (1.12, 1.14) |
| | Aged 50-64 | 70,408 | 6363 | -3.3% | 0.87 (0.86, 0.88) | -21.9% | 0.75 (0.74, 0.76) | 73,872 | 8097 | 0.8% | 1.02 (1.01, 1.03) | -8.6% | 1.04 (1.03, 1.05) |
| **Anxiety disorders** | **Sex** | | | | | | | | | | | | |
| | Females | 76,454 | 3736 | -3.0% | 0.86 (0.86, 0.87) | -19.4% | 0.78 (0.78, 0.79) | 77,633 | 4793 | 0.5% | 1.03 (1.02, 1.04) | -7.2% | 1.14 (1.12, 1.15) |
| | Males | 45,649 | 2750 | -3.9% | 0.85 (0.83, 0.86) | -14.9% | 0.81 (0.80, 0.82) | 47,891 | 3638 | 0.5% | 1.03 (1.01, 1.04) | 0.9% | 1.13 (1.12, 1.15) |
| | Male/Female Ratio[f] | | | | 0.74 (0.73, 0.74) | | 0.74 (0.73, 0.74) | | | | 0.76 (0.75, 0.77) | | 0.76 (0.75, 0.77) |
| | **Age** | | | | | | | | | | | | |
| | Aged 18-24 | 17,422 | 3066 | -6.4% | 0.83 (0.81, 0.85) | -13.2% | 0.85 (0.83, 0.87) | 17,738 | 4086 | 3.7% | 1.05 (1.03, 1.07) | -0.5% | 1.24 (1.21, 1.27) |
| | Aged 25-49 | 70,168 | 3443 | -2.8% | 0.85 (0.85, 0.86) | -17.5% | 0.79 (0.78, 0.80) | 71,179 | 4466 | -0.5% | 1.02 (1.01, 1.03) | -4.4% | 1.13 (1.12, 1.14) |
| | Aged 50-64 | 34,873 | 3151 | -3.1% | 0.87 (0.86, 0.88) | -20.3% | 0.76 (0.75, 0.77) | 36,983 | 4054 | 1.2% | 1.03 (1.01, 1.04) | -5.6% | 1.07 (1.06, 1.09) |
| **Depressive disorders** | **Sex** | | | | | | | | | | | | |
| | Females | 41,597 | 2033 | -7.1% | 0.83 (0.82, 0.84) | -33.1% | 0.65 (0.64, 0.66) | 45,973 | 2839 | 1.0% | 1.03 (1.02, 1.04) | -17.2% | 1.01 (1.00, 1.03) |
| | Males | 30,923 | 1863 | -3.7% | 0.85 (0.83, 0.86) | -27.1% | 0.69 (0.68, 0.70) | 33,312 | 2531 | 1.3% | 1.04 (1.02, 1.05) | -12.7% | 0.98 (0.96, 0.99) |
| | Male/Female Ratio[f] | | | | 0.92 (0.90, 0.93) | | 0.92 (0.90, 0.93) | | | | 0.89 (0.88, 0.90) | | 0.89 (0.88, 0.90) |
| | **Age** | | | | | | | | | | | | |
| | Aged 18-24 | 10,814 | 1903 | -9.5% | 0.80 (0.78, 0.82) | -22.9% | 0.75 (0.74, 0.77) | 12,213 | 2814 | 7.8% | 1.09 (1.07, 1.12) | -11.1% | 1.11 (1.08, 1.13) |
| | Aged 25-49 | 37,328 | 1831 | -4.2% | 0.84 (0.83, 0.85) | -31.5% | 0.66 (0.65, 0.66) | 40,385 | 2534 | 0.4% | 1.03 (1.02, 1.05) | -16.2% | 0.99 (0.98, 1.00) |
| | Aged 50-64 | 24,532 | 2217 | -6.2% | 0.84 (0.83, 0.86) | -32.3% | 0.65 (0.64, 0.66) | 26,865 | 2945 | -0.5% | 1.01 (0.99, 1.03) | -16.0% | 0.95 (0.94, 0.97) |
| **Bipolar disorders** | **Sex** | | | | | | | | | | | | |
| | Females | 16,650 | 814 | -5.3% | 0.84 (0.83, 0.86) | -27.7% | 0.70 (0.69, 0.72) | 17,872 | 1103 | 1.6% | 1.04 (1.02, 1.06) | -12.1% | 1.07 (1.05, 1.10) |
| | Males | 12,688 | 764 | -7.4% | 0.81 (0.80, 0.83) | -23.0% | 0.73 (0.71, 0.75) | 14,051 | 1068 | 3.1% | 1.05 (1.03, 1.08) | -3.8% | 1.08 (1.05, 1.10) |
| | Male/Female Ratio[f] | | | | 0.94 (0.92, 0.96) | | 0.94 (0.92, 0.96) | | | | 0.97 (0.95, 0.99) | | 0.97 (0.95, 0.99) |
| | **Age** | | | | | | | | | | | | |

| | | | | | | | | | | | | | |
|---|---|---|---|---|---|---|---|---|---|---|---|---|---|
| | Aged 18-24 | 3,562 | 627 | -6.1% | 0.83 (0.80, 0.87) | -19.0% | 0.79 (0.76, 0.83) | 3,870 | 892 | 6.1% | 1.07 (1.03, 1.12) | -6.8% | 1.16 (1.11, 1.21) |
| | Aged 25-49 | 17,282 | 848 | -6.2% | 0.82 (0.81, 0.84) | -26.3% | 0.71 (0.69, 0.72) | 18,825 | 1181 | 2.6% | 1.05 (1.03, 1.08) | -9.0% | 1.08 (1.06, 1.10) |
| | Aged 50-64 | 8,616 | 779 | -6.0% | 0.84 (0.82, 0.87) | -26.9% | 0.70 (0.68, 0.72) | 9,315 | 1021 | 0.1% | 1.01 (0.99, 1.04) | -8.9% | 1.04 (1.01, 1.06) |
| | | | | | | | | | | | | | |
| **Schizophrenia spectrum disorders** | **Sex** | | | | | | | | | | | | |
| | Females | 8,819 | 431 | -4.4% | 0.85 (0.83, 0.88) | -19.1% | 0.79 (0.77, 0.81) | 9,570 | 591 | 3.3% | 1.05 (1.02, 1.08) | -0.8% | 1.21 (1.18, 1.25) |
| | Males | 16,415 | 989 | -7.3% | 0.82 (0.80, 0.83) | -13.7% | 0.82 (0.80, 0.83) | 18,474 | 1404 | 1.2% | 1.03 (1.01, 1.06) | 8.5% | 1.22 (1.19, 1.24) |
| | Male/Female Ratio[f] | | | | 2.29 (2.24, 2.35) | | 2.29 (2.24, 2.35) | | | | 2.38 (2.32, 2.43) | | 2.38 (2.32, 2.43) |
| | **Age** | | | | | | | | | | | | |
| | Aged 18-24 | 2,214 | 390 | -8.1% | 0.82 (0.77, 0.86) | -16.5% | 0.82 (0.77, 0.86) | 2,487 | 573 | 3.1% | 1.04 (0.99, 1.10) | 1.7% | 1.27 (1.20, 1.34) |
| | Aged 25-49 | 15,534 | 762 | -6.2% | 0.82 (0.81, 0.84) | -12.4% | 0.84 (0.82, 0.86) | 17,157 | 1076 | 1.6% | 1.04 (1.02, 1.07) | 9.4% | 1.29 (1.27, 1.32) |
| | Aged 50-64 | 7,561 | 683 | -6.2% | 0.84 (0.82, 0.87) | -21.1% | 0.76 (0.73, 0.78) | 8,463 | 928 | 2.1% | 1.04 (1.00, 1.07) | -1.6% | 1.12 (1.09, 1.15) |
| | | | | | | | | | | | | | |
| **Trauma and stressor-related disorders** | **Sex** | | | | | | | | | | | | |
| | Females | 10,757 | 526 | -8.6% | 0.82 (0.79, 0.84) | -26.3% | 0.72 (0.70, 0.74) | 11,514 | 711 | 5.5% | 1.08 (1.05, 1.11) | -5.7% | 1.15 (1.12, 1.18) |
| | Males | 8,116 | 489 | -3.3% | 0.85 (0.83, 0.88) | -22.7% | 0.73 (0.71, 0.75) | 8,794 | 668 | 4.1% | 1.06 (1.03, 1.10) | -1.4% | 1.11 (1.07, 1.14) |
| | Male/Female Ratio[f] | | | | 0.93 (0.90, 0.96) | | 0.93 (0.90, 0.96) | | | | 0.94 (0.91, 0.97) | | 0.94 (0.91, 0.97) |
| | **Age** | | | | | | | | | | | | |
| | Aged 18-24 | 2,871 | 505 | -14.5% | 0.76 (0.72, 0.80) | -24.3% | 0.74 (0.71, 0.78) | 3,292 | 758 | 7.0% | 1.08 (1.03, 1.14) | -1.4% | 1.23 (1.17, 1.29) |
| | Aged 25-49 | 11,231 | 551 | -4.6% | 0.84 (0.82, 0.86) | -24.9% | 0.72 (0.70, 0.74) | 11,830 | 742 | 2.7% | 1.06 (1.03, 1.08) | -6.3% | 1.11 (1.08, 1.14) |
| | Aged 50-64 | 4,880 | 441 | -4.8% | 0.85 (0.82, 0.89) | -24.5% | 0.72 (0.70, 0.75) | 5,281 | 579 | 8.7% | 1.10 (1.06, 1.15) | 0.4% | 1.14 (1.10, 1.18) |
| | | | | | | | | | | | | | |
| **Attention-deficit/hyperactivity disorders** | **Sex** | | | | | | | | | | | | |
| | Females | 2,956 | 144 | -9.9% | 0.80 (0.76, 0.84) | -30.1% | 0.68 (0.65, 0.71) | 3,078 | 190 | 4.7% | 1.07 (1.02, 1.12) | -16.9% | 1.02 (0.97, 1.07) |
| | Males | 3,091 | 186 | -5.3% | 0.83 (0.79, 0.87) | -31.0% | 0.65 (0.62, 0.68) | 3,219 | 245 | 1.6% | 1.04 (0.99, 1.09) | -11.3% | 0.99 (0.95, 1.04) |
| | Male/Female Ratio[f] | | | | 1.29 (1.23, 1.36) | | 1.29 (1.23, 1.36) | | | | 1.29 (1.22, 1.35) | | 1.29 (1.22, 1.35) |
| | **Age** | | | | | | | | | | | | |
| | Aged 18-24 | 1,935 | 341 | -11.4% | 0.79 (0.74, 0.84) | -34.3% | 0.64 (0.61, 0.68) | 2,046 | 471 | 5.4% | 1.07 (1.00, 1.14) | -19.6% | 1.00 (0.94, 1.06) |
| | Aged 25-49 | 3,346 | 164 | -6.1% | 0.83 (0.79, 0.87) | -31.5% | 0.66 (0.63, 0.69) | 3,551 | 223 | 2.8% | 1.06 (1.01, 1.11) | -11.5% | 1.05 (1.00, 1.10) |
| | Aged 50-64 | 797 | 72 | -2.8% | 0.87 (0.79, 0.96) | -13.1% | 0.83 (0.76, 0.92) | 740 | 81 | 0.5% | 1.02 (0.92, 1.13) | -9.8% | 1.03 (0.93, 1.13) |
| | | | | | | | | | | | | | |
| **Disruptive behavioral and impulse disorders** | **Sex** | | | | | | | | | | | | |
| | Females | 920 | 45 | 4.7% | 0.93 (0.85, 1.02) | -10.6% | 0.87 (0.80, 0.95) | 886 | 55 | -4.9% | 0.97 (0.89, 1.06) | -1.0% | 1.21 (1.10, 1.33) |
| | Males | 1,969 | 119 | -4.1% | 0.84 (0.79, 0.90) | -15.7% | 0.80 (0.75, 0.85) | 2,234 | 170 | 5.0% | 1.07 (1.01, 1.14) | 14.2% | 1.28 (1.20, 1.36) |
| | Male/Female Ratio[f] | | | | 2.64 (2.44, 2.85) | | 2.64 (2.44, 2.85) | | | | 3.10 (2.87, 3.35) | | 3.10 (2.87, 3.35) |
| | **Age** | | | | | | | | | | | | |
| | Aged 18-24 | 618 | 109 | -11.5% | 0.79 (0.71, 0.88) | -29.6% | 0.69 (0.62, 0.76) | 760 | 175 | 11.8% | 1.13 (1.02, 1.26) | 7.5% | 1.34 (1.21, 1.48) |
| | Aged 25-49 | 1,762 | 86 | 4.0% | 0.91 (0.86, 0.98) | -4.7% | 0.91 (0.86, 0.97) | 1,795 | 113 | -0.1% | 1.03 (0.96, 1.10) | 12.9% | 1.34 (1.25, 1.43) |
| | Aged 50-64 | 518 | 47 | -5.5% | 0.85 (0.75, 0.96) | -20.6% | 0.76 (0.68, 0.85) | 576 | 63 | -2.4% | 0.99 (0.88, 1.11) | 2.5% | 1.16 (1.04, 1.31) |
| | | | | | | | | | | | | | |
| **Obsessive-compulsive disorders** | **Sex** | | | | | | | | | | | | |
| | Females | 684 | 33 | -7.2% | 0.83 (0.75, 0.92) | -25.1% | 0.73 (0.66, 0.80) | 729 | 45 | 6.4% | 1.09 (0.98, 1.21) | 1.5% | 1.24 (1.12, 1.38) |
| | Males | 582 | 35 | -3.0% | 0.85 (0.76, 0.96) | -21.8% | 0.74 (0.66, 0.83) | 631 | 48 | -0.2% | 1.02 (0.91, 1.14) | 18.8% | 1.33 (1.19, 1.50) |
| | Male/Female Ratio[f] | | | | 1.05 (0.94, 1.17) | | 1.05 (0.94, 1.17) | | | | 1.07 (0.96, 1.18) | | 1.07 (0.96, 1.18) |

© 2022 Anderson KN et al. *JAMA Psychiatry.*

| | | | | | | | | | | | | | | | | | | |
|---|---|---|---|---|---|---|---|---|---|---|---|---|---|---|---|---|---|---|
| | **Age** | | | | | | | | | | | | | | | | | |
| | Aged 18-24 | 223 | 39 | | -18.6% | 0.72 (0.61, 0.86) | | -25.7% | 0.73 (0.61, 0.86) | | 250 | 58 | | -4.9% | 0.96 (0.81, 1.15) | | -7.8% | 1.15 (0.97, 1.36) |
| | Aged 25-49 | 723 | 35 | | -0.6% | 0.87 (0.79, 0.97) | | -24.0% | 0.73 (0.66, 0.80) | | 775 | 49 | | 8.4% | 1.11 (1.01, 1.23) | | 12.8% | 1.33 (1.20, 1.48) |
| | Aged 50-64 | 330 | 30 | | -6.5% | 0.84 (0.72, 0.97) | | -20.5% | 0.76 (0.66, 0.88) | | 348 | 38 | | 0.9% | 1.02 (0.88, 1.19) | | 19.2% | 1.35 (1.16, 1.58) |
| **Eating disorders** | **Sex** | | | | | | | | | | | | | | | | | |
| | Females | 608 | 30 | | -12.9% | 0.78 (0.70, 0.87) | | -13.6% | 0.84 (0.75, 0.94) | | 712 | 44 | | 6.1% | 1.08 (0.97, 1.20) | | 12.7% | 1.38 (1.24, 1.53) |
| | Males | 149 | 9 | | -6.3% | 0.82 (0.66, 1.03) | | -6.9% | 0.88 (0.71, 1.10) | | 153 | 12 | | 10.9% | 1.13 (0.90, 1.43) | | 4.8% | 1.17 (0.94, 1.47) |
| | Male/Female Ratio[f] | | | | | 0.30 (0.25, 0.36) | | | 0.30 (0.25, 0.36) | | | | | | 0.26 (0.22, 0.31) | | | 0.26 (0.22, 0.31) |
| | **Age** | | | | | | | | | | | | | | | | | |
| | Aged 18-24 | 245 | 43 | | -17.2% | 0.73 (0.62, 0.87) | | -5.4% | 0.92 (0.78, 1.10) | | 334 | 77 | | 12.5% | 1.14 (0.97, 1.33) | | 22.8% | 1.53 (1.30, 1.79) |
| | Aged 25-49 | 375 | 18 | | -8.8% | 0.80 (0.70, 0.92) | | -15.5% | 0.81 (0.71, 0.93) | | 397 | 25 | | 5.0% | 1.08 (0.94, 1.24) | | 2.9% | 1.22 (1.06, 1.40) |
| | Aged 50-64 | 141 | 13 | | -10.2% | 0.81 (0.64, 1.01) | | -13.5% | 0.83 (0.66, 1.04) | | 138 | 15 | | 0.0% | 1.01 (0.80, 1.28) | | 11.3% | 1.26 (0.99, 1.61) |
| **Tic disorders** | **Sex** | | | | | | | | | | | | | | | | | |
| | Females | 109 | 5 | | -18.1% | 0.73 (0.57, 0.94) | | 9.0% | 1.06 (0.81, 1.39) | | 122 | 8 | | 25.8% | 1.28 (0.98, 1.68) | | 34.1% | 1.64 (1.25, 2.15) |
| | Males | 152 | 9 | | -21.2% | 0.69 (0.56, 0.86) | | -15.6% | 0.80 (0.64, 0.99) | | 153 | 12 | | -3.8% | 0.98 (0.79, 1.23) | | 0.7% | 1.13 (0.90, 1.41) |
| | Male/Female Ratio[f] | | | | | 1.72 (1.34, 2.20) | | | 1.72 (1.34, 2.20) | | | | | | 1.54 (1.22, 1.96) | | | 1.54 (1.22, 1.96) |
| | **Age** | | | | | | | | | | | | | | | | | |
| | Aged 18-24 | 91 | 16 | | -19.5% | 0.71 (0.54, 0.94) | | 13.8% | 1.11 (0.82, 1.50) | | 104 | 24 | | 60.0% | 1.62 (1.19, 2.21) | | 73.3% | 2.16 (1.57, 2.97) |
| | Aged 25-49 | 119 | 6 | | -21.2% | 0.69 (0.55, 0.88) | | -20.7% | 0.76 (0.60, 0.97) | | 139 | 9 | | -8.0% | 0.95 (0.75, 1.19) | | -4.8% | 1.13 (0.89, 1.42) |
| | Aged 50-64 | 53 | 5 | | -17.2% | 0.74 (0.52, 1.07) | | 3.9% | 1.00 (0.68, 1.46) | | 33 | 4 | | -19.5% | 0.82 (0.52, 1.29) | | -21.4% | 0.89 (0.57, 1.41) |

[a]NSSP collects free-text reason for visit (chief complaint), discharge diagnosis, and patient demographic details. Diagnosis information is collected using International Classification of Diseases, Ninth Revision, Clinical Modification (ICD-9-CM) codes; International Classification of Diseases, Tenth Revision, Clinical Modification (ICD-10-CM) codes; and Systematized Nomenclature of Medicine (SNOMED) codes. Emergency department visits associated with any MH-related visit and specific mental disorders were identified by querying ED visits data from the National Syndromic Surveillance Program (NSSP) using keyword syndromes developed by CDC in partnership with state and local health departments (eTable 1); [b]To reduce artifactual impact from changes in reporting patterns, analyses examining trends in MH-related ED visits by sex and age were restricted to facilities with a coefficient of variation ≤40 and average weekly discharge diagnosis ≥75% from January 2019–August 2021. [c]Rate of ED visits for MH outcome = (number of ED visits for MH outcome/number of total ED visits in the time period of interest for the subpopulation of interest) x 100,000; [d]Percent change in visits was calculated as the difference in total visits between the index period and the comparison period, divided by the total visits during the comparison period, times 100 ([ED visits for MH outcome during index period–ED visits for MH outcome during comparison period]/ED visits for MH outcome during comparison period*100%); [e]Visit ratios = Proportion ED visits for MH outcome during the index period, divided by the proportion of ED visits for MH outcome during the comparison period ([ED visits for MH outcome {index period}/all ED visits {index period}]/(ED visits for MH outcome {comparison period}/all ED visits {comparison period}]); [f]Male to female visit ratios = (proportion of ED visits for MH outcome during index period for males/proportion of ED visits for MH outcome during index period for females). Ratios >1 indicate a higher proportion of ED visits for MH outcome during the surveillance period for males compared with females.

© 2022 Anderson KN et al. *JAMA Psychiatry.*

**eAppendix 1.** Detailed Footnotes for Figure 2 Mental Health–Related, Anxiety, and Depressive Disorder US Emergency Department Visits, by Race and Ethnicity

**U.S.=United States; ED=emergency department. This figure includes data from December 27, 2020 through August 14, 2021. Shaded areas of the graph depict time periods included in analytic index or comparison periods. From left to right, this includes a COVID-19 case peak comparison period from Dec. 27, 2020–Jan. 23, 2021; the period immediately following a COVID-19 case peak index period of Feb. 14, 2021–Mar. 13, 2021; a COVID-19 pandemic comparison period with low circulation of the Delta variant (Pre-Delta period): Apr. 18–May 15, 2021; and a COVID-19 pandemic index period with high circulation of the Delta variant (Delta period): July 18–Aug. 14, 2021. These windows were used in the temporal comparisons displayed in Table 2. Comparisons between racial and ethnic groups for ED visit rates for each MH outcome of interest were non-temporal and within the index period of interest. In the Delta period, prevalence ratios ≥1.0 with 95% confidence intervals excluding 1.0 indicated that there were the following differences between groups: (1) non-Hispanic White persons had higher prevalence of any MH-related ED visits compared to non-Hispanic American Indian or Alaska Native, non-Hispanic Asian, Hispanic, non-Hispanic Black, and non-Hispanic Native Hawaiian or Other Pacific Islander persons; (2) non-Hispanic American Indian or Alaska Native persons had higher prevalence of any MH-related ED visits compared to non-Hispanic Black, Hispanic, and non-Hispanic Native Hawaiian or Other Pacific Islander persons; (3) non-Hispanic White persons had higher prevalence of anxiety disorder-related ED visits compared to non-Hispanic American Indian or Alaska Native, non-Hispanic Asian, Hispanic, non-Hispanic Black, and non-Hispanic Native Hawaiian or Other Pacific Islander persons; (4) non-Hispanic American Indian or Alaska Native persons had higher prevalence of anxiety disorder-related ED visits compared to non-Hispanic Black and non-Hispanic Native Hawaiian or Other Pacific Islander persons; (5) non-Hispanic Black persons had lower prevalence of anxiety disorder-related ED visits compared to non-Hispanic Asian and Hispanic persons; (6) non-Hispanic White persons had higher prevalence of depressive disorder-related ED visits compared to non-Hispanic American Indian or Alaska Native, non-Hispanic Asian, non-Hispanic Black, and Hispanic persons; (7) non-Hispanic American Indian or Alaska Native persons had higher rates of depressive disorder-related ED visits compared to non-Hispanic Black and Hispanic persons; and (8) non-Hispanic Asian persons had higher prevalence of depressive-disorder related ED visits compared to Hispanic persons. No other comparisons between racial and ethnic groups showed differences. In the period after a COVID-19 case peak, prevalence ratios ≥1.0 with 95% confidence intervals excluding 1.0 comparing between racial and ethnic groups showed similar findings as to the Delta period with several exceptions: (1) non-Hispanic White persons showed no differences in prevalence of MH-related ED visits with non-Hispanic American Indian or Alaska Native persons; (2) non-Hispanic American Indian or Alaska Native persons had higher prevalence of any MH-related ED visits compared to non-Hispanic Asian persons; (3) non-Hispanic American Indian or Alaska Native persons had higher prevalence of anxiety disorder-related ED visits compared to Hispanic and non-Hispanic Asian persons; (4) non-Hispanic American Indian or Alaska Native persons had higher prevalence of depressive disorder-related ED visits compared to non-Hispanic Asian persons; (5) non-Hispanic Asian persons showed no differences in prevalence of depressive disorder-related ED visits compared to Hispanic persons; (6) non-Hispanic White persons showed no differences in prevalence of depressive disorder-related ED visits compared to non-Hispanic American Indian or Alaska Native persons.**

© 2022 Anderson KN et al. *JAMA Psychiatry.*

**eAppendix 2.** Detailed Footnotes for Figure 3 Bipolar, Schizophrenia Spectrum, and Trauma- and Stressor-Related Disorder US Emergency Department Visits, by Race and Ethnicity

**U.S.=United States; ED=emergency department. This figure includes data from December 27, 2020 through August 14, 2021. Shaded areas of the graph depict time periods included in analytic index or comparison periods. From left to right, this includes a COVID-19 case peak comparison period from Dec. 27, 2020–Jan. 23, 2021; the period immediately following a COVID-19 case peak index period of Feb. 14, 2021–Mar. 13, 2021; a COVID-19 pandemic comparison period with low circulation of the Delta variant (Pre-Delta period): Apr. 18–May 15, 2021; and a COVID-19 pandemic index period with high circulation of the Delta variant (Delta period): July 18–Aug. 14, 2021. These windows were used in the temporal comparisons displayed in Table 2. Comparisons between racial and ethnic groups for ED visit rates for each MH outcome of interest were non-temporal and within the index period of interest. In the Delta period, prevalence ratios ≥1.0 with 95% confidence intervals excluding 1.0 indicated that there were the following differences between groups: (1) non-Hispanic White persons had higher prevalence of bipolar disorder-related ED visits compared to non-Hispanic Black, non-Hispanic Asian, Hispanic, and non-Hispanic American Indian or Alaska Native persons; (2) non-Hispanic Black persons had higher prevalence of bipolar disorder-related ED visits compared to non-Hispanic American Indian or Alaska Native and Hispanic persons; (3) non-Hispanic White persons had higher prevalence of schizophrenia spectrum disorder-related ED visits compared to Hispanic persons; (4) non-Hispanic Black persons had higher prevalence of schizophrenia spectrum disorder-related ED visits compared to non-Hispanic American Indian or Alaska Native, non-Hispanic Asian, Hispanic, and non-Hispanic White persons; (5) non-Hispanic American Indian or Alaska Native persons had higher prevalence of trauma and stressor-related disorder ED visits compared to non-Hispanic Asian, Hispanic, and non-Hispanic Black persons; (6) non-Hispanic White persons had higher prevalence of trauma and stressor-related disorder ED visits compared to non-Hispanic Black persons. No other comparisons between racial and ethnic groups showed differences. In the period after a COVID-19 case peak, prevalence ratios ≥1.0 with 95% confidence intervals excluding 1.0 comparing rates between racial and ethnic groups showed similar findings as to the Delta period with several exceptions: (1) non-Hispanic Black persons showed no differences in prevalence of bipolar disorder-related ED visits with non-Hispanic American Indian or Alaska Native persons; (2) non-Hispanic American Indian or Alaska Native persons had higher prevalence of trauma and stressor-related disorder ED visits compared to non-Hispanic White persons; (3) non-Hispanic White persons had higher prevalence of trauma and stressor-related disorder ED visits compared to Hispanic and non-Hispanic Asian persons.**

© 2022 Anderson KN et al. *JAMA Psychiatry.*
